# Sleep Disturbances in Children Affected by Autism Spectrum Disorder

**DOI:** 10.3389/fpsyt.2022.736696

**Published:** 2022-02-17

**Authors:** Jessica Galli, Erika Loi, Lucrezia Maria Visconti, Paola Mattei, Anna Eusebi, Stefano Calza, Elisa Fazzi, ASD Collaborative Group, Luisa Bonini

**Affiliations:** ^1^Department of Clinical and Experimental Sciences, University of Brescia, Brescia, Italy; ^2^Unit of Child Neurology and Psychiatry, ASST Spedali Civili of Brescia, Brescia, Italy; ^3^Department of Molecular and Translational Medicine, University of Brescia, Brescia, Italy; ^4^BDbiomed, BODaI Lab, University of Brescia, Brescia, Italy

**Keywords:** autism spectrum disorder, sleep disturbance, insomnia, parent stress, melatonin

## Abstract

**Background:**

Sleep disturbances are common in children affected by Autism Spectrum Disorder (ASD). The aim of our study was to describe sleep characteristics and disturbances in children with ASD, to evaluate possible related factors, and to assess parental stress.

**Methods:**

Hundred children with a diagnosis of ASD (mean age: 66.7 months, SD: 27.4, range: 24.7–152.1 months, *n* = 79 males) were included in the study. We collected data on sociodemographic, clinical, genetic and instrumental variables as well as comorbid conditions. Parents filled out the Questionnaire on sleep behavior in the first years of life, the BEARS questionnaire, and the Parenting Stress Index Short Form. From the analysis on sleep characteristics, we excluded 25 children treated with melatonin.

**Results:**

Fifty-seven (57%) out of 100 children met the criteria for insomnia. Sleep disorders were associated with developmental or cognitive delay, emotional and behavioral problems (such as anxiety problems and aggressive behaviors) and absence of strategies for inducing sleep after nocturnal awakenings. From parents' reports, sleep disorders had diurnal repercussions on their offspring; however, we found no statistical correlation between disturbances and family stress. Also, no significant correlation was found between sleep disturbances and epilepsy. Finally, a statistical correlation was found between the regular intake of melatonin and the resolution of insomnia.

**Conclusions:**

Multifactorial variables may be associated to insomnia that could have an impact on the children' behavior. Clinicians need to be aware of the value of screening for sleep disturbance in children with ASD to integrate sleep interventions in the treatment plan.

## Introduction

Autism spectrum disorder (ASD) is a life-long neurodevelopmental condition affecting communication and social interaction across multiple contexts and it includes the presence of atypical and heterogeneous behaviors such as restricted and repetitive patterns of interests or activities manifesting with different degrees of severity; intellectual disability or global developmental delay does not explain it any better (1). Sleep disorders are frequently observed in children with ASD with a higher prevalence (50–80%) (2) compared to that observed in typically developing children (25–40%) (3) and in children affected by other neurodevelopmental disabilities [Down syndrome, 55% or cerebral palsy, 43% (4)]. The type of sleep disturbances is similar in children with and without neurodevelopmental disorders (5). The most described insomnia (6) is onset insomnia or difficulty initiating sleep and sleep maintenance. Besides insomnia, parasomnia such as sleepwalking, nightmare, pavor nocturnus and sleep apnea, enuresis, difficult awakening and daytime sleepiness are also reported (7, 8).

Although children with ASD seem to have a greater vulnerability to sleep problems, the pathophysiology of these difficulties remains unclear. Mazzone et al. (9) offered three hypotheses. First of all, sleep problems may be explained as the consequence of neurobiological and genetic alteration that result in sleep architecture disruption; for instance, abnormal expressions of several neurotransmitters implicated in sleep regulation [such as serotonin, melatonin (10–15), and gamma-aminobutyric acid] (16, 17), and mutations in circadian-relevant genes as NR1D1, CLOCK, ARNTL2 (10), PER1-2-3 (18) have been reported in subjects with ASD. Second, sleep problems may themselves be a core aspect of ASD related to the specific characteristics of the autism. For example, not adhering to bed time rituals, excessive and repetitive activities before bed-time, communication deficits, hypo- or hyper-arousal, sensory dysregulation (19, 20), food selectivity, phobias (21), medical comorbidities and pharmacological treatment as risperidone (9, 22) may cause delayed sleep onset or insomnia. Third, sleep problems could be co-occurring disorders independent from ASD. In addition to the above specific mechanisms, physiological stressors such as being sick, psychological aspects as a difficult day at school (23), child rearing practices (24), and poor sleep hygiene behavior have been suggested as non-specific causes of sleep disturbances.

Sleep disruption may negatively act at neuroanatomical and clinical levels. Research on animals and on typically developing children demonstrated that sleep deprivation may lead to impairments of adequate synaptic development and brain maturation (24, 25); therefore, some authors have speculated that sleep dysfunction in children with ASD could actively cause disorders in synaptic function, potentially creating a maladaptive feed-forward loop of sleep disruption and neural anomalies (24, 25). Furthermore, less sleeping time and worse quality of sleep may affect adaptive behavior (e.g., hygiene, eating, toileting…), cognition, attention, memory, learning (26–30) and executive functioning (31), and increase internalizing and externalizing problems, as depression, anxiety, self-injury, oppositional behavior, irritability, physical aggression (24, 32–35). Sleep problems may also exacerbate the core symptom severity of ASD: poor sleepers were reported to show more restricted and stereotyped behaviors, more severe social skills deficits (2, 26, 36–40) and communication problems (41). However, it is still unclear what daytime implications of sleep disorder are and the direction of their relationship.

Parenting a child with ASD is *per se* a heavy burden related to emotional, behavioral and communication difficulties (42). In addition, child sleep problems may further increase the family distress affecting the quality of parental sleep (43) and exacerbating offspring symptoms, particularly behavioral problems such as hyperactivity, self-injury, aggressiveness (9).

From a holistic perspective, to our knowledge, there is no study that considers sleep in terms of sleep characteristics (duration, habits and rituals, awakenings), sleep disturbances (insomnia, breathing related sleep disorders or parasomnias) and their impact on child and family life. The aims of our study were: (1) to detail the characteristics of sleep in children with ASD and the type of their sleep disturbances; (2) to evaluate any possible factors (related to ASD condition itself, as severity of autism and comorbidities, and to sleep habits) associated with sleep disturbances; (3) to assess the parents' perception of the sleep disorder of their ASD offspring and whether sleep disturbance has a negative impact on children behavior and parenting stress; (4) to explore the effect of melatonin on sleep disturbances in a small subgroup of ASD children.

## Materials and Methods

### Participants

We carried out an observational study of 100 children with ASD (mean age: 66.7 months, SD: 27.4, range: 24.7–152.1 months, *n* = 79 males) recruited at the Unit of Child and Adolescent Neurology and Psychiatry of ASST Spedali Civili of Brescia, Italy, between January 2020 and January 2021. We got a written informed consent from parents and we conducted the study according to the Declaration of Helsinki which was approved by the Institutional Review Board of ASST Spedali Civili of Brescia (Comitato Etico di Brescia, ID number 4085 SLEEPASD). Participation was voluntary, and the data were processed anonymously.

The diagnosis of autism was the only inclusion criterion, previously made in accordance with the Diagnostic and Statistical Manual of Mental Disorders, 5th Edition (DSM-V) (1) criteria and performed by a multidisciplinary team including a child neuropsychiatrist and an experienced child psychologist.

We collected sociodemographic data as: age and sex of the patient, family structure (siblings: number, sex, age, health condition; parents: age, nationality, marital status, education, and occupation) and family socioeconomic status (SES). The following data on clinical and genetic/instrumental characteristics were obtained from charts: pregnancy, delivery, gestational age, neurological examination (normal or pathological according to the absence/presence of one or more abnormalities concerning cranial nerve, muscle strength, tone and bulk, reflexes and gait), pharmacological treatment, genetic analysis (karyotype, FRAXA, CGH-array), and instrumental examination (brain magnetic resonance imaging and electroencephalogram) defined as “normal” or “altered” in the absence/presence of genetic, structural brain and/or electroencephalographic abnormalities.

We performed the Autism Diagnostic Observation Schedule, 2nd Edition (ADOS-2) (44) to determine the autism symptom severity in 79 children. We collected data on comorbidities as developmental/cognitive delay, emotional and behavioral difficulties and epilepsy. It is noteworthy that epilepsy has been reported to define in children with ASD a different subgroup in terms of clinical characteristics including intellectual disability (45).

Finally, parents filled out three questionnaires at home on: sleep characteristics (duration, habits, rituals and awakenings), sleep disturbances, and parental stress levels. After completion, parents returned the questionnaires during any programmed visits to our Unit. We carried out a diagnosis of sleep disturbances (insomnia, breathing related sleep disorders, or parasomnias) using the findings from the questionnaires and according to the DSM-V criteria.

To avoid a possible bias related to pharmacological intake of hypnotic drugs, we split the total sample in two subgroups: 75 children were not treated with hypnotic drugs and included in the analyses on sleep characteristics, and 25 were treated with melatonin, excluded from the mentioned analyses. In this subgroup, we evaluated the persistence of sleep disturbances after the beginning of the treatment.

### Assessments

#### Family Socioeconomic Status

We measured family SES by the Hollingshead Scale (Hollingshead, unpublished manuscript)^1^, which ranks social position based on maternal and paternal occupation and educational attainment. The occupation scores range from 1 to 9 and the education scores from 1 to 7, with lower scores reflecting lower occupational/educational status. We considered the items (Occupation score × 5, Education score × 3) and summed them to produce an Index of Social Position Score (range: 8–66) classified into five categories, with higher scores indicating higher status.

#### Autism Symptoms Severity

The Autism Diagnostic Observation Schedule 2nd edition (ADOS-2) (44) is a semi-structured, standardized assessment of communication, social interaction, play and restricted and repetitive behaviors. It consists of five modules selected based on the expressive language level and the chronological age and comprises two domains: Social Affect and Restricted Repetitive Behavior. Calibrated Severity Scores were calculated to yield 1 of 3 clinical severity levels: low level of autistic symptoms, medium level, high level.

#### Developmental Profile/Cognitive Level

Developmental profile was evaluated in 81 children using the Griffiths Mental Development Scales Extended Revised (GMDS-ER) (46). It examines six domains of functioning, individually scored: Locomotor, Personal-Social, Hearing and Speech, Eye and Hand Coordination, Performance and Practical Reasoning. A Total Score called Developmental Quotient (DQ) is obtained from the average of the five domains. All the quotients are reported in standard scores (DQ: mean 100, SD 15). Children performances were rated as follows: more than −1 DS, normal; between −1 and −2 DS, borderline; < -2 DS, delay.

Full Intelligence Quotient was evaluated in 19 children using age-appropriate versions of Wechsler intelligence scales IV edition (47). The quotient is reported in standard scores (mean 100, SD 15) rated as follows: more than −1 DS, normal; between −1 and −2 DS, borderline; < -2 DS, delay.

#### Emotional and Behavioral Difficulties

To evaluate the risk of emotional and behavioral difficulties, the parent report Child Behavior Checklist 1½-5 (48, 49) or 6–18 (50) was carried out according to age. The item scores are aggregated in: (1) three composite scales (Internalizing, Externalizing, and Total problems); (2) syndrome scales (CBCL 1½-5: Emotionally reactive, Anxious/Depressed, Somatic complaints, Withdrawn, Sleep problems, Attention problems, and Aggressive behavior; CBCL 6–18: anxious/depressed, withdrawn/ depressed, somatic complaints, social problems, thought problems, attention problems, rule-breaking behavior and aggressive behavior); (3) DSM-oriented scales (CBCL 1½-5: Affective problems, Anxiety problems, Pervasive developmental problems, Attention deficit/Hyperactivity problems, and Oppositional defiant problems; CBCL 6-18: affective problems, anxiety problems, somatic problems, attention deficit/hyperactivity problems, oppositional defiant problems, conduct problems). T scores are created based on a normative sample; the cut-off points were categorized as “normal,” “borderline,” or “clinically significant” according to the tool kit software standards.

#### Sleep Characteristics and Disturbances

##### Questionnaire on Sleep Behavior in the First Years of Life

The questionnaire (51) elicits assessable information about sleep behavior. It is structured in a series of single answers, multiple and forced choice (YES/NO) questions except for the last part that allows parents to express freely their own opinions on their child's sleep behavior. We chose to administer this questionnaire because it provides: first, sociodemographic data (age, gender, nationality, marital status, education level, and occupation) of the parents and on the family structure; second, a deep and detailed description of sleep behavior since the first years, considering also habits, rituals and any form of strategies adopted by parent to favor sleep; third, information on parents' habits (as waking up during the night to check their baby) and perception of their child sleep (as problematic or not).

##### BEARS Questionnaire

The BEARS (52) is a screening tool developed to address the most common sleep issues in toddlers, preschoolers, and school aged children (2–18 year old age range). Five sleep domains are evaluated through a set of age-appropriate “trigger questions”. The five sleep domains are: Bedtime Problems, including difficulty going to bed and falling asleep; Excessive Daytime Sleepiness, which includes behaviors typically associated with daytime somnolence in children; Awakenings during the night; Regularity of sleep/wake cycles (bedtime, wake time) and average sleep duration; Snoring. We chose to use the BEARS questionnaire in order to support the findings obtained from the Questionnaire on sleep behavior in the first years of life because it is a brief and easy tool used to screen sleep problems systematically. Owens and Dalzell (53) in their pilot study demonstrated the usefulness of the questionnaire when compared with the usual consultation because of the increased amount of information on children sleep behavior and problems.

#### Parental Stress

The Parenting Stress Index Short Form (PSI-SF) (54), one of the most widely used tools for measuring parenting stress, was used. It contains 36 items divided into three subscales: “Parental distress” refers to the distress felt by the parent in his/her parental role, “Parent-child dysfunctional interaction” indicates the level of satisfaction perceived by the parent in the relationship with the offspring, and “Difficult child” specifies to the degree of difficulty experienced by the parent regarding the child's behavior. Each item is rated from 1 (strongly disagree) to 5 (strongly agree). The PSI-SF gives three subscores and a total score. Higher scores (at 90th percentile) are associated with significant parental stress level.

### Statistical Analysis

Once the questionnaires were returned, we did statistical analyses using R statistical package (version 4.0.3) setting the statistical significance threshold at 5%. We described the qualitative variables as number and percentage and the quantitative variables as mean, standard deviation and range. We applied Pearson's Chi-squared test to study the relationship between sleep disturbances and sociodemographic (age, sex, pregnancy and delivery, gestational age, and family variables), clinical (neurological examination and severity of ASD), and genetic/instrumental data. In addition, we investigated the relationship between sleep disturbances and comorbidities, sleep characteristics, and parental stress index.

## Results

In Tables 1, 2, respectively, we report sociodemographic data and children demographic, clinical, genetic and instrumental characteristics; while Table 3 shows comorbid conditions of the sample.

**Table 1:** Sociodemographic data and family structure.

	**Mothers (*N* = 100)**	**Fathers (*N* = 100)**
Mean age in years ± SD (range)	37.8 ± 5.8 (23-50)	41.3 ± 7.1 (25-66)
Nationality, *N* (%)		
Italians	54 (54)	63 (63)
Foreigners	46 (46)	37 (37)
Level of education, *N* (%)		
Elementary school	1 (1)	1 (1)
Middle school	19 (19)	23 (23)
Vocational or high school	55 (55)	57 (57)
Bachelor's degree	20 (20)	15 (15)
Master's and/or PhD	5 (5)	4 (49)
Employment status, *N* (%)		
Unemployed	51 (51)	5 (5)
Employed	49 (49)	95 (95)
Marital status, *N* (%)		
Single	11 (11)	11 (11)
Married	87 (87)	87 (87)
Divorced	2 (2)	2 (2)
	**Family**	
Mean SES scores ± SD (range)	30.4 ± 11.6 (8–66)	
Lower status	16	
Lower-middle status	43	
Middle status	23	
Upper-middle status	14	
Upper status	4	
	**Total sample (*****N*** **=** **100)**	
Siblings		
*N*, mean age in months ± SD (range)	89, 106.5 ± 72.9 (4–288)	
Male/female distribution, *N* (%)	51 (57) / 38 (43)	
Siblings with neurological disorder/ASD	6/8	

*N, number; SD, standard deviation; SES, socioeconomic status; ASD, autism spectrum disorder*.

**Table 2:** Demographic, clinical, and genetic/instrumental data of the total sample.

	**Total sample (*****N*** **=** **100)**	
Mean age in months ± SD (range)	66.7 ± 27.4 (24.7–152.1)	
Males/females, *N* (%)	79 (79) / 21 (21)	
Fertilization		
Natural, *N* (%)	95 (95)	
*In vitro, N* (%)	3 (3)	
Intracytoplasmic sperm injection, *N* (%)	2 (2)	
Pregnancy		
Uneventful, *N* (%)	72 (72)	
Pathological, *N* (%)	28 (28)	
Delivery		
Vaginal, *N* (%)	64 (64)	
Scheduled/urgent cesarean, *N* (%)	22 (22) / 14 (14)	
Preterm birth, *N* (%)	9 (9)	
Mean gestational age (weeks) ± SD (range)	39 ± 2.1 (28–42)	
Birth weight (grams) ± SD (range)	3195.8 ± 605.4 (1124–4600)	
Neurological examination		
Sign and symptoms of ASD	100 (100)	
Hypotonia/macrocrania	23 (23) / 6 (6)	
ADOS-2^*^, calibrated severity score		
Low level	10 (12)	
Medium level	40 (51)	
High level	29 (37)	
Therapy		
Melatonin	25 (25)	
Antiepileptic drugs	3 (3)	
Risperidone	2 (2)	
**Genetic analysis**	**Normal**	**Altered**
Karyotype (performed in 53 cases), *N* (%)	53 (100)	0 (0)
FRAXA (performed in 53 cases), *N* (%)	53 (100)	0 (0)
CGH-array (performed in 56 cases), *N* (%)	38 (68)	18 (32)
**Instrumental examination**		
Brain MRI (performed in 27 cases), *N* (%)	18 (67)	9 (33)
EEG (performed in 56 cases), *N* (%)	39 (70)	17 (30)

*N, number; SD, standard deviation; ASD, autism spectrum disorder; MRI, magnetic resonance imaging; EEG, electroencephalogram*.

* *Carried out in 79 children*.

**Table 3:** Comorbidities of the sample.

	**Total sample (*****N*** **=** **100)**			**Children not treated (*N* = 75)**
**Developmental/cognitive delay, *N* (%)**	**Normal**	**Borderline**	**Delay**	**Insomnia**
Developmental quotient, (*N* = 81)	1 (1)	8 (10)	72 (89)	*p* = 0.01
Full intelligence quotient, (*N* = 19)	10 (53)	6 (31)	3 (16)	
**Emotional-behavioral problems**, ***N*** **(%)**	**Normal**	**Borderline**	**C. significant**	
**CBCL 1½-5 and 6–18 composite scales**				
Total problems	52	11	37	*p* <001
Internalizing problems	50	15	35	*p* <0.01
Externalizing problems	70	7	23	*p* <0.01
**CBCL syndrome scales**				
CBCL 1½−5 (*N* = 62)				
Emotionally reactive	36 (58)	10 (16)	16 (26)	*p* <0.01
Anxious/depressed	46 (74)	5 (8)	11 (18)	*p* <0.05
Somatic complaint	51 (82)	6 (10)	5 (8)	
Withdrawn	30 (48)	9 (14)	23 (37)	
Aggressive behavior	52 (84)	5 (8)	5 (8)	*p* <0.01
Attention problems	41 (66)	4 (7)	17 (27)	
Sleep problems	50 (81)	7 (11)	5 (8)	*p* <0.01
CBCL 6-18 (*N* = 38)				
Anxious/depressed	38 (100)	0	0	
Withdrawn	12 (31)	23 (61)	3 (8)	
Somatic complaints	37 (97)	1 (3)	0	
Social problems	30 (79)	7 (18)	1 (3)	*p* <0.01
Thought problems	17 (45)	11 (29)	10 (26)	
Attention problems	13 (34)	7 (18)	18 (48)	
Rule-breaking behavior	33 (87)	1 (3)	4 (10)	*p* <0.01
Aggressive behavior	31 (82)	2 (5)	5 (13)	*p* <0.01
**CBCL DSM-oriented scales**				
CBCL 1½−5 (*N* = 62)				
Affective problems	42 (68)	12 (19)	8 (13)	*p* <0.01
Anxiety problems	49 (79)	1 (2)	12 (19)	*p* <0.01
Pervasive developmental problems	13 (21)	16 (26)	33 (53)	
Attention deficit/hyperactivity problems	52 (84)	9 (14)	1 (2)	
Oppositional defiant problems	50 (81)	9 (14)	3 (5)	*p* <0.05
CBCL 6–18 (*N* = 38)				
Affective problems	29 (76)	6 (16)	3 (8)	
Anxiety problems	31 (81)	1 (3)	6 (16)	*p* <0.05
Somatic problems	32 (84)	6 (16)	0	*p* <0.05
Attention deficit/hyperactivity problems	18 (47)	14 (37)	6 (16)	
Oppositional defiant problems	29 (76)	7 (18)	2 (6)	
Conduct problems	32 (84)	0	6 (16)	*p* <0.01
**Epilepsy**, ***N*** **(%)**	3 (3)			

*N, number; C, clinically*.

### Sleep Characteristics in Children With ASD Not Treated With Melatonin (75 Subjects)

Table 4 shows the findings of the Questionnaire on sleep behavior in the first years of life regarding sleep duration, habits and rituals, nocturnal awakenings and waking up time, and it shows the BEARS questionnaire in the 75 subjects not treated with melatonin.

**Table 4:** Sleep characteristics according to the two sleep questionnaires.

	**Children not treated (*****N*** **=** **75)**	
	***N* (%)**	**Insomnia**
**Questionnaire on sleep behavior in the first years of life**		
**Average hours of night-time sleep**		
<7; between 7 and 9; between 10 and 12; >12 h	2 (3); 24 (32); 48 (64); 1 (1)	
**Average hours of daytime sleep**		
<2; ≥2 h	9 (32); 19 (68)	
**Sleep-time habits and rituals**		
Put to bed at regular time/when experience fatigue	64 (85)/11 (15)	
Put to bed by the same person [mother; father]	43 (57) [38 (88); 5 (12)]	
Has a ritual for getting to sleep:	65 (87)	
Presence of a parent nearly to the bed/in the same bed	34 (45) / 24 (32)	
Telling stories; singing lullabies	19 (25); 8 (11)	
Presence of a light on; door open	7 (9); 1 (1)	
Drinking chamomile, tea or milk	8 (11)	
Watching electronical devices; listening to music	2 (3); 2 (3)	
Being rocked in the bed/arms	6 (8) / 2 (3)	
Utilizing transitional objects; using baby pacifier	4 (5); 3 (4)	
Mean time for falling asleep in minutes ± SD (range)	22 ± 21.9 (5–90)	
Sleeps with parents [same room/same bed]; with siblings	41 (55) [9 (22)/32 (78)]; 11 (15)	
Sleep position: on one side/supine or prone/genupectoral	21 (28) / 16 (21) / 2 (3)	
Moves a lot during sleep/throws off the covers	28 (37) / 52 (69)	
**Nocturnal awakenings (NA)**		
Children with NA	46 (61)	
Mean of NA per night ± SD (range)	1.7 ± 1.2 (1–5)	
Mean of nights with NA per week ± SD (range)	4.7 ± 1.5 (1–6)	
**Duration of NA:**		
<5; between 5 and 15; 16 and 30; 31 and 60; >60 min	22 (29) / 7 (9) / 7 (9) / 2 (3) / 8 (11)	
All the night [frequency: < once a month; every night]	5 (7) [4 (80); 1 (20)]	
One or more actions: cries; plays alone; does nothing	11 (15); 4 (5); 17 (22)	
Parents adopting strategies for inducing sleep	22 (29)	*p* <0.01
Parents wake up to check on the child	40 (53)	
**Waking up time**		
Wakes up spontaneously/needs to be waked up	45 (60) / 30 (40)	
Has waking up rituals (kissing, cuddling, hugging)	26 (35)	
At awakening child seems to be: calm; lively; very lively	54 (72); 20 (27); 1 (1)	
**Parents' perception of the offspring sleep disturbances**		
Perception of sleep as problematic	17 (23)	*p* <0.01
Diurnal repercussion	12 (16)	*p* <0.01
**BEARS questionnaire**		
Bedtime problems	48 (64)	
Excessive daytime sleepiness	28 (37)	*p* <0.01
Awakenings during the night	13 (17)	
Irregularity of sleep/wake cycle	12 (16)	
Snoring	14 (19)	

*N, number; SD, standard deviation*.

Data collected from both questionnaires helped us to diagnose insomnia, according to DSM-V criteria, in 32 (43%) children: 17 subjects (53%) had difficulty in sleep onset, 5 (16%) difficulty in maintaining sleep due to frequent awakenings or problems returning to sleep after awakenings and 10 (31%) both disturbances. None of the children in our sample presented Breathing-Related Sleep Disorders or Parasomnias.

### Factors Related to Sleep Disturbances

No sociodemographic data (age, sex, pregnancy, delivery, gestational age, and family variables) of children with ASD seemed to be correlated with the presence of insomnia, as well as the characteristics of neurological examination, the severity of ASD and the genetic/instrumental data.

Correlations between comorbid conditions and insomnia are summarized in Table 3. Specifically, we observed a statistically significant association between sleep disorder and a developmental or cognitive delay (*p* = 0.01). As regards emotional and behavioral difficulties, considering both CBCL 1½>-5 and CBCL 6–18, statistical correlations were found between the presence of insomnia and the occurrence of internalizing, externalizing, and total problems (*p* < 0.01). Analyzing the syndrome scales, a statistically significant correlation was detected between sleep disturbance and an increased risk of emotionally reactive problems (*p* < 0.01), anxious/depressed problems (*p* < 0.05), aggressive behavior and sleep problems (*p* < 0.01) at CBCL 1½-5, and social problems, rule-breaking behavior, and aggressive behavior (*p* < 0.01) at CBCL 6–18. Moreover, insomnia correlated with the following DSM-oriented scales: affective problems (*p* < 0.01), anxiety problems (*p* < 0.01), and oppositional defiant problems (*p* < 0.05) at CBCL 1½-5, and anxiety problems (*p* < 0.05), somatic problems (*p* < 0.05), and conduct problems (*p* < 0.01) at CBCL 6–18. Epilepsy did not correlate with sleep disturbances.

Different sleep-time habits and rituals used both by the children themselves and by parents to favor the sleep or places where children sleep during the night did not correlate with the presence or absence of insomnia. However, we found a statistical correlation between the use of strategies and rituals adopted to induce sleep after nocturnal awakenings and the back to sleep (*p* < 0.01): in fact, children that did not have sleep rituals presented difficulties in going back to sleep.

### Parents' Perception of the Sleep Disorder and Their Level of Stress

According to the questionnaire on sleep behavior in the first years of life, 58 (77%) parents did not perceive their offspring sleep as problematic (in 40 cases there was not a sleep disturbance); the remaining 17 (23%) parents judged it as problematic (in 14 cases insomnia was confirmed). A statistically significant association between the presence of insomnia and parents' perception of sleep as problematic was found (*p* < 0.01). Diurnal repercussions, reported in 12 children (insomnia detected in 10 of them), correlated with the sleep disturbance (*p* < 0.01): in fact, children with insomnia seemed to be more aggressive and nervous during daytime activities. BEARS questionnaire also confirmed that children with insomnia seemed to be sleepier during the day (*p* < 0.01). The above correlations are reported in Table 4.

The PSI-SF revealed normal results in 60 (80%) cases, borderline in 5 (7%) and pathological in 10 (13%) at Total Stress Scale. At “Parental Distress” subscale we observed normal scores in 69 (92%) parents, borderline in 1 (1%) and pathological in 5 (7%); at “Parent-Child Dysfunctional Interaction” subscale normal results in 59 (78%) parents, borderline in 5 (7%), and 11 (15%) pathological; and at “Difficult Child” subscale normal scores in 57 (76%) parents, borderline in 7 (9%) and pathological in 11 (15%). No statistical correlation was found between sleep disturbances and PSI-SF total score and each subscale.

### Sleep Disturbances in Children Treated With Melatonin (25 Subjects)

As declared in section Materials and Methods we evaluated the persistence of insomnia (already diagnosed at the time of the enrollment) in 25 subjects treated with melatonin (1–3 mg/die, 30 min before falling asleep at about 8.30 p.m., for at least 3 months) to induce sleep. At the time of the questionnaire, insomnia persisted in 10 (40%) cases, 8 (80%) of them assumed melatonin occasionally while 2 (20%) in association with risperidone. The remaining 15 (60%) children took regularly (every night) melatonin without other drugs and they did not present sleep disturbances after the beginning of the treatment. Comparing children who assumed melatonin with regularity and those who took it occasionally, we found a statistical correlation between the regular intake of the drug and the resolution of insomnia (*p* < 0.01).

## Discussion

The present study arises from the need to describe the sleep characteristics (duration, habits and rituals, and nocturnal awakenings) and the presence of a sleep disturbance in children affected by ASD, to evaluate any possible associated factors, parents' perception of the sleep disorder, and any possible consequences of sleep problems on the child and the family. Finally, we also explored the effect of melatonin on insomnia in a small subgroup of ASD children.

Our study confirms that insomnia is a hallmark of sleep disorder in ASD. Considering children treated with melatonin and those without any hypnotic drug, more than half (57 children, 57%) of our sample suffered from insomnia, specifically sleep onset problems and sleep settling issues, due to frequent awakenings or difficulties returning to sleep after awakenings; our data are in line with those of literature (2, 5, 55, 56). However, we did not observe other sleep disturbances, such as sleep-related breathing and movement disorders probably due to difficulties in making a diagnosis using questionnaires without polysomnography (56).

In accordance with literature (8), night sleep duration was between 9.0 and 12.00 h and the most common habits and rituals involved the presence of parents during the falling asleep phase and nocturnal awakenings in a higher percentage compared to literature (8). The reasons for co-sleeping in ASD subjects are different: firstly, the rate of sleep disturbances is high and consequently this increases the likelihood of wanting to sleep with parents. Moreover, parents themselves may think that the child needs more assistance during the night and therefore they may prefer to sleep with their child; finally the repetitive and limited behaviors characterizing ASD itself could prevent changing this habit (8). In our study there is no evidence that co-sleeping improves the sleep of children; it may be useful to implement behavioral therapies teaching parents the correct strategies to keep toward their children, for example avoiding responding to the child's disruptive bedtime behaviors (crying, calling out, and leaving the bedroom) as well as checking on the child, but only when he/she is showing the desired behaviors (quiet, calm, and in his/her bed) (57). No specific characteristics in bedtime routine or in the rituals performed during the night correlated with the absence of insomnia in our study; hence every behavioral therapy should be personalized keeping in mind the individuality of each child, and needs and preferences of parents, in order to create a balanced routine for the family.

Among comorbid conditions, developmental/cognitive delay and emotional/behavioral difficulties were associated with insomnia. The association between sleep disturbances and cognitive functioning is not well established because some studies reported an increase of sleep difficulties in children with ASD and cognitive deficit (2) while others the occurrence regardless of the cognitive functioning (58). Moreover, the direction of this relationship needs to be clarified (59): do sleep problems cause cognitive deficits or *vice versa*? Recent researches hypothesized that sleep is essential for optimizing cognitive development, memory and learning (31, 58) and that chronic sleep deprivation has negative effects on cognitive and academic performance (31, 41) probably influencing cerebral connectivity and creating aberrant neural pathways (60).

According to the literature (9, 32), our study revealed that children with insomnia were more likely to exhibit internalizing (as anxious/depressed problems), externalizing (aggressive/social problems and rule-breaking behavior) and total behavior difficulties compared to those without it. Anxiety and mood disorders may be associated with hyperarousal, intrusive thoughts and worries during the pre-sleep time, leading to difficulties in falling asleep and insomnia (9) and at the same time sleep loss may affect behavioral regulation and determine daytime fatigue (32), predisposing to aggression, property destruction (35) and violence (61). The association observed here in children with ASD between insomnia and aggression is of particular interest given the associations found by several authors between aggression and endogenous opioids and steroids in animal models, knowing that abnormalities of the opioid and steroid systems have been reported in ASD (62, 63). This suggests that there is a vulnerability for aggression in ASD, and insomnia may major this vulnerability for aggression or result from it.

In contrast with literature (64), epilepsy did not correlate with sleep disturbances; a possible explanation may be the limited number of subjects affected by epilepsy enrolled in the study.

Sleep rituals and strategies seemed to favor the back to sleep after a nocturnal awakening. According to literature data, sleep problems observed in ASD population may be related also to inadequate “sleep hygiene” (parenting related factors associated with the promotion of the child's sleep) (65), such as inconsistent bedtime routine, delayed or irregular bedtimes, access to electronic media devices (65). Although the exact mechanisms of sleep hygiene practices are still unclear (66), establishing some behavioral conditions, as the reduction of external stimulation, the increase in relaxation, and regular bedtime routine with little variability over nights may play an important role in favoring sleep (7).

In our study, parent's perception of their offspring sleep (judged as “problematic” or “not problematic”) correlated with the diagnosis of insomnia. Moreover, parents reported that sleep disorders have diurnal repercussions as being more aggressive and nervous during activities and excessive daily somnolence; this data confirms other previous findings (32, 67, 68). Interventions addressed to limit sleep disturbances in subjects with ASD improve behavioral problems (69, 70); hence, considering parents' perceptions and experiences of their children' sleep provides insightful information for the clinicians and it helps to identify additional factors that may contribute to insomnia and reduce the efficacy of interventions (71).

In contrast to our hypothesis, poor sleep did not affect parental mental health since no statistical differences were found between the stress levels of parents of children with and without insomnia. Conversely, literature documents that sleep disturbances in children with ASD increase parenting burden and family stress (72). An explanation could be related to the possibility of having developed resilience and adaptive coping strategies to manage difficulties parents face every day, reducing the perception of family distress (73). In fact, less than a third of parents reported being stressed. Another possible explanation could be the fact that parents did not answer authentically to the questionnaire aimed at investigating their challenges, because too focused on problems experienced by their child.

Although the high frequency of insomnia in our sample, only a small number of children took melatonin to favor sleep. The night-time administration of melatonin seemed to lead to improvements of the sleep disturbance in more than half of our cases, but its efficacy was observed in all the children that assumed it every day and without other medications. Melatonin appears to be useful for treatment of sleep disturbances in ASD (56), however its potential mechanism on poor sleep is not completely understood (74, 75). It may help to regulate the circadian rhythm, to act as sedative/hypnotic agent or to replace the melatonin deficiency observed in some ASD children (76). Melatonin may also contribute to reduce the levels of antioxidants (75, 77), particularly high in some children with ASD, and to regulate the synaptic plasticity, altered by an imbalance in excitatory and inhibitory systems (72–76).

The absence of any comparison group and of normative data for the two sleep questionnaires administered may represent potential limitations of the present study. We are aware of the difficulty to evaluate the clinical significance of our findings; however, we made diagnosis of insomnia according to the DSM-V criteria and we assessed comorbid conditions using standardized tests according to normative parameters.

In summary, the results from this study suggest that children affected by ASD showed high prevalence of sleep disturbances, in particular insomnia, which seemed to be associated with developmental/cognitive delay, emotional and behavior problems and poor sleep hygiene, without increasing the family stress. Regular melatonin administration may ameliorate sleep disturbances. As congruence, a screening for sleep disturbance in children with ASD is necessary to integrate sleep interventions in the treatment plan.

## Data Availability Statement

The raw data supporting the conclusions of this article will be made available by the authors, without undue reservation.

## Ethics Statement

The studies involving human participants were reviewed and approved by Comitato Etico di Brescia, ID Number 4085. Written informed consent to participate in this study was provided by the participants' legal guardian/next of kin.

## ASD Collaborative Group

Luisa Bonini, Angela Merlini, Laura Pansera, Laura Malerba, and Filippo Gitti, Unit of Child Neurology and Psychiatry, ASST Spedali Civili of Brescia, Brescia, Italy; Silvia Saottini, Department of Clinical and Experimental Sciences, University of Brescia, Brescia, Italy.

## Author Contributions

JG, EL, and LV wrote the article. LV, PM, and AE collected data and administered the sleep questionnaires. JG, PM, and EF designed the study. SC performed the statistical analysis. All authors contributed to the article, reviewed the manuscript, and approved the submitted version.

## Funding

Università degli Studi di Brescia provides fees for open access publication.

## Conflict of Interest

The authors declare that the research was conducted in the absence of any commercial or financial relationships that could be construed as a potential conflict of interest.

## Publisher's Note

All claims expressed in this article are solely those of the authors and do not necessarily represent those of their affiliated organizations, or those of the publisher, the editors and the reviewers. Any product that may be evaluated in this article, or claim that may be made by its manufacturer, is not guaranteed or endorsed by the publisher.

## Footnotes

^1^Hollingshead AB. *Four factor index of social status*. (1975) Unpublished manuscript, Yale University, New Haven, CT.
